# What Motivates People to Pay for Online Sports Streaming? An Empirical Evaluation of the Revised Technology Acceptance Model

**DOI:** 10.3389/fpsyg.2021.619314

**Published:** 2021-05-28

**Authors:** Ye Sun, Huifeng Zhang

**Affiliations:** The School of Journalism and Communication, Renmin University of China, Beijing, China

**Keywords:** livestreaming, sports, perceived enjoyment, willingness to pay, technology acceptance model

## Abstract

With the rapid development of Internet technology, sports media consumption is migrating toward streaming. The online streaming of sporting events has become the important way of copyrights holders to engage sports fans, especially young fans. Therefore, it is vital to understanding users' willingness to pay (WTP). Given that previous studies of the livestream sports broadcasts online have not dealt with users' payment intention, the originality of this study is that it explore users' motivation by combining information system research with the consumer demand theory. The study aimed to examine the factors that influence users' payment intention to stream online sports by using the extended Technology Acceptance Model (TAM). Data from questionnaires completed by 330 Chinese respondents determined how perceived usefulness, ease, enjoyment, and satisfaction, influence users' WTP. Satisfaction positively correlated with users' payment intentions, impacted WTP values, mediated ease of use. The analysis also revealed the necessity for broadcast platforms to improve satisfaction and to increase payment intentions.

## Introduction

Consumers expect copyright owners to use various online platforms to distribute media effectively and to their smart-phones, laptops PCs, and other wireless streaming devices. It is clear that the COVID-19 further accelerate the digital transformation of sports industry. Due to postponement and cancellation of sports events, COVID-19 has deprive spectators, pushing copyrights owners to reshape and augment the digital users' experience in order to generate more revenue from online sports streaming. According to the survey of top opportunities to increase revenues in the sports industry, 89.3% of the respondents consider that enhancing digital media fan experience is the best way (PwC, 2020). Online media are expected to be a significant source of revenue in the coming years (Liu and Peng, 2017). In contrast, livestream sports platforms face cyber risk, such data risk, cyber-crime and human error (Radanliev et al., 2020; Sheehan et al., 2021). To improve the experience of watching the sports events on the network platform, the online sports streaming medias adopt artificial intelligence technology, but it neglects the security of users' data and aggravate the cyber risk, which can easily lead to the leakage of users' data (Radanliev et al., 2020). Moreover, owing to the anonymity of the “bullet screen” service of the online sports streaming media, cyber-criminal organizations use the “bullet screen” to induce users to illegal websites, resulting in users' financial losses. Based on this, the development of network video platform has both opportunities and challenges. And there is a gap between the number of viewers who currently pay and those who do not. Hence, it is necessary to analyze the motivations of paying users.

Most studies on sports media subscriptions and subscribers have regarded the marketing strategy of online platforms (Evens et al., 2011; Hao, 2019), though few studies have focused on users' willingness to pay. This study extends the research scope from offline to online, from public sports programs to commercial sports media. From the perspective of influencing factors, recent researches about public sports program have shown about public that consumers' payment intentions are determined by two kinds of variables, individual characteristic variables (e.g., age, income, gender), psychological variables (e.g., identification, trust, satisfaction) (see Table 1). However, technology readiness level that have not been explored in the previous study is essential to network live streaming media. This study attempts to explored technology readiness level and other internal motivational factors that affect users' willingness to pay (WTP) for online sports streaming.

**Table 1:** Contributions of previous Johnson authors.

**References**	**Research subject**	**Influencing factors**		
		**Demographic variables**	**Psychological variables**	**Technical variables**
Johnson et al. (2007)	Amateur sport programs	YES	e.g., moral norms	NA
Wicker (2011)	Non-profit sports clubs	YES	NA	NA
Bakkenbüll and Dilger (2018)	World cup success	YES	e.g., identification, personal importance, national importance	NA
Funahashi and Mano (2015)	Elite sport policy	YES	e.g., benefit perception, risk perception, Trust, knowledge	NA
Wicker et al. (2016)	Sporting success	NA	e.g., happy, reputation, talking about the team	NA
Zawadzki (2016)	Mega sports events	YES	e.g., national pride, intangible costs	NA
Frick and Wicker (2017)	World championships	YES	e.g., expectations, consumption capital, happy	NA
Wicker et al. (2017)	Mega sports events	YES	e.g., identify, proud, happy, reputation, role models, interest	NA
Wicker et al. (2018)	Sports clubs	YES	e.g., identification, satisfaction	NA
De Boer et al. (2019)	Mega sports events	YES	NA	NA
Wicker and Frick (2020)	Elite athlete development	YES	e.g., life satisfaction, identification, role models, happy	NA
Thormann and Wicker (2021)	Non-profit sport clubs	YES	e.g., environmental consciousness, well-being	NA
This Paper	Online sports streaming	YES	e.g., satisfaction, perceived enjoyment	e.g., perceived usefulness, perceived ease of use

*NA, Not Applicable. The variables are not mentioned in the paper*.

Reviewing the previous literatures, it was found that WTP has been employed to study consumers' payment intention, while TAM has been utilized to explain the influential factors of users' behavioral intentions. Thus, combining the TAM with WTP can answer the question, “What motivates people pay for online sports events?” Two external variables about technology readiness level in the original TAM, perceived usefulness and perceived ease of use as related to payment behavioral intention (Arogundade et al., 2016; Vladova et al., 2021), but few prior studies confirmed internal variables, such as perceived enjoyment as bearing influence on a consumer's willingness to pay (Joeckel and Bowman, 2012).

Congruent with these previous studies, this study evaluates the relationship between these two external variables and users' willingness to pay. The present research introduces two internal variables—perceived enjoyment and perceived satisfaction—to further examine the determinants of consumers' willingness to pay for live sports online. The new evaluation model is appropriate for identifying the factors affecting users' willingness to pay to view sports matches online and provides valuable marketing information to the online platforms for developing their own digital media distribution strategy to satisfy consumer demands.

## Theoretical Background and Prior Study

### Theory of the Technology Acceptance Model

The technology acceptance model (TAM) is the most frequently adopted theory in explaining technology usage (Princi and Krämer, 2020). Due to the simple construction of TAM, it has been widely adopted to investigate users' acceptance of different kinds of technologies.

Adapted from the theory of reasoned action (TRA), TAM was introduced by Davis (1989) to specifically explain the behavior of users' technology acceptance. In the original TAM, five specific variables, including perceived usefulness, perceived ease of use, attitude toward using, behavioral intention to use, and actual system use, were factors developed to explain technology acceptance. Both perceived usefulness and perceived ease of use, were the dominant determinants of users' attitudes toward using new technology (Davis et al., 1989). The TAM also suggested that perceived usefulness and users' attitude toward directly and significantly influence behavioral intention to use (Davis et al., 1989). In addition, it posits that users' actual use of a new system or technology is mainly determined by behavioral intention. Understanding why people accept or reject a new system or technology has been one of the most popular subjects in recent research (Abdullah et al., 2016; Lemay et al., 2018). However, prior studies have produced no reliable evidence to indicate a significant relationship between perceived usefulness and an attitude toward using a new technology (Rafique et al., 2020; Lin et al., 2021). Congruent with these studies, the attitude was removed from the original TAM to confirm the relationship between external variables (e.g., perceived usefulness and perceived ease of use) and behavioral intention to use in the present research (Rauniar et al., 2014; Abed, 2016). Instead, we added an intrinsic motivation variable—perceived enjoyment—to develop a more convincing explanation of what might affect the behavioral intention of the hedonic system (Dickinger et al., 2008; Vladova et al., 2021). In addition, satisfaction was introduced to TAM as a direct determinant of behavioral intention.

The purpose of the TAM is to provide a scientific, rational, and efficient model to explain what motivates users to accept or reject a new technology. Based on the previous theoretical and empirical literature, this study adds two new variables—perceived enjoyment and satisfaction—to the research model in order to quantify users' willingness to pay for livestream sports broadcast platforms.

### Previous Study of the Willingness to Pay

Willingness to pay (WTP) originated in the study of economic impacts on consumers. WTP suggests the value of a product or service to a consumer is equivalent to the maximum price a consumer is willing to pay for that product or service (Kalish and Nelson, 1991). In recent studies, WTP theory has been applied to public goods and commercialized goods, including sports teams (Wicker et al., 2016), sporting events (Li and Yang, 2019), and media products (Zhang and Deng, 2018).

According to WTP, two factors determine the extent to which consumers are willing to pay for products or services. The first factor of WTP investigates the consumer's payment intention. The second factor of WTP considers consumer's WTP value in terms of the consumer's willingness to pay a premium price (Tudoran and Olsen, 2017). Integrating the TAM into the evaluation of consumer motivation has advanced researchers' abilities to study the factors that influence consumers' willingness to pay (Arogundade et al., 2016).

In regard to WTP, payment intention acts as behavioral intention in the modified TAM. However, the variable of actual use can be replaced by the WTP value (Arogundade et al., 2016). Consistent with the findings of the original TAM (Zhao et al., 2020), the results of prior studies confirmed that perceived usefulness and perceived ease of use are significant determinants of payment intention. Besides, payment intention has a significant effect on the WTP value (Wicker et al., 2016).

Understanding why consumers are willing to pay for livestream sports broadcasts is essential for both providers and consumers. This study integrates the processes of the TAM and WTP model to establish an integrated model that can explain the factors that determine users' payment intention and result in a WTP value for livestream sports broadcasts.

## Hypotheses and Research Model

### Perceived Usefulness

Drawing on the theory of reasoned action, perceived usefulness and perceived ease of use are regarded as the fundamental determinants of user behavior in the TAM (Davis et al., 1989). Streaming media is considered as a new and efficient service that could save users' time and effort (Alalwan et al., 2018). Thus, perceived usefulness is re-defined as “which a person believes that using streaming media devices would improve his or her working/living quality” (Yang and Lee, 2018).

A series of previous studies indicated that perceived usefulness is positively associated with behavioral intention (Persico et al., 2014; Kumar Kakar, 2017; Chen and Wu, 2020). Perceived usefulness mediates the relationship between perceived ease of use and intention (Park et al., 2007; Carranza et al., 2021). Based on the empirical support from prior research, this present study seeks to revalidate similar relationships in the context of payment for livestream sports subscriptions. Hence, the following hypotheses are proposed:

**H1**. Perceived usefulness will have a positive effect on users' intentions to purchase livestream sports broadcasts online.
**H1A**. Perceived usefulness will mediate the effect of perceived ease of use on users' intention to purchase livestream sports broadcasts online.

### Perceived Ease of Use

The TAM emphasizes the role of perceived ease of use in behavioral intentions to use different kinds of systems. Perceived ease of use, in its original form, refers to “the degree to which a person believes that using a particular system would be free of effort (Davis et al., 1989).” Base on the definition, Users' perceived ease of use for streaming media refers to the degree to which users can easily operate the devices (Yang and Lee, 2018). It could be more complicated for users to watch sports events by streaming media than by traditional TV (Alalwan et al., 2018; Yang and Lee, 2018). A number of previous studies showed that perceived ease of use significantly and positively affected users' intentions (Alalwan et al., 2016; Zhao et al., 2020). Hence, the following hypothesis is proposed:

**H2**. Perceived ease of use will have a positive effect on users' payment intention to livestream sports broadcasts online.

According to the TAM, perceived ease of use is directly linked to usefulness because the easier the system is to use, the more useful it can be (Davis et al., 1989). There is extensive research on various systems that provides evidence of the significance of perceived ease of use on perceived usefulness (Rauniar et al., 2014; Alalwan et al., 2016). Congruent with the previous studies of the original TAM, this study proposes the following hypothesis:

**H2A**. Perceived ease of use will have a positive effect on the perceived usefulness of livestream sports broadcasts online.

As an intrinsic motive, perceived enjoyment is influenced by perceived ease of use. Based on the definition of perceived ease of use, when the system is easy to use, users require less mental effort to use it, which may stimulate users' emotional feedback, ultimately increasing their subjective enjoyment of interacting with the system (Wang and Scheepers, 2012). As with the relationship between perceived ease of use and perceived usefulness, the easier the system is to use, the more enjoyable it can be (Van der Heijden, 2003). The results of numerous empirical studies provided evidence that perceived ease of use is an essential factor in determining users' enjoyment of using systems (Dickinger et al., 2008; Abed, 2016). As such, this study proposes:

**H2B**. Perceived ease of use will have a positive effect on perceived enjoyment of the livestream sports broadcasts online.

Drawing on the relationship between perceived ease of use and perceived usefulness, the easier the system is to use, the more satisfied users can be (Rahmi et al., 2018). Previous studies indicated that a positive relationship exists between perceived ease of use and satisfaction (Nagy, 2018). Hence, the following hypothesis is proposed:

**H2C**. Perceived ease of use will have a positive effect on users' satisfaction with livestream sports broadcasts online.

### Perceived Enjoyment

To examine the effect of users' intrinsic motivation on technology acceptance, the TAM model introduces the concept of perceived enjoyment (Davis et al., 1992; Van der Heijden, 2003). Base on the previous study, perceived enjoyment in this study is defined as the extent to which watching livestream sports is perceived as enjoyable in its own right, apart from any performance consequence that may be anticipated (Abdullah et al., 2016). Perceived enjoyment becomes a key factor underlying user acceptance of hedonic systems.

Some studies conceived perceived enjoyment as an intrinsic motivation and tested the effect of perceived enjoyment on hedonic system usage, with products such as websites, online video platforms, and social media (Wang and Scheepers, 2012; Jung et al., 2018). These studies confirmed the mediating effect between perceived ease of use and behavioral intention of perceived enjoyment (Van der Heijden, 2003). The results confirmed that the intention to use a hedonic system is mostly influenced by enjoyment (Dickinger et al., 2008; Abed, 2016; Moghavvemi et al., 2017). Congruent with the previous literature, this study posited the following hypotheses:

**H3**. Perceived enjoyment will have a positive effect on the payment intention for livestream sports broadcasts online.
**H3A**. Perceived enjoyment will mediate the effect of perceived ease of use on users' intention to livestream sports broadcasts online.

### Satisfaction

Marketing and customer psychology have developed the foundation for the philosophy in marketplace satisfaction. Satisfaction is defined as a rate to which products are able to meet customer expectations (Oliver, 1981). In addition, satisfaction is highly based on emotion (Liao et al., 2009; Lee and Lehto, 2013). In this study, satisfaction refers to the degree to which the actual performance of the livestream broadcast meets users' expectations. Significantly, satisfaction can be measured only after using the system or technology (Nagy, 2018).

A number of previous studies have prorated the TAM to apply satisfaction to previous research (Alraimi et al., 2015; Amin et al., 2020) and affirmed the effect of satisfaction on behavioral intention, either directly or indirectly, through perceived ease of use (Dehghan et al., 2012; Alraimi et al., 2015). Hence, the following hypotheses are proposed:

**H4**. Satisfaction will have a positive effect on users' payment intention to livestream sports broadcasts online.
**H4A**. Satisfaction will mediate the effect of perceived ease of use on users' intention to purchase or subscribe to livestream sports broadcasts online.

### Willingness to Pay

Willingness to pay is described as a customer's inclination to pay the maximum amount for a product or service (Tudoran and Olsen, 2017). There are two stages of willingness to pay, including payment intention and WTP values. If products or services satisfy customers' intentions and the price is lower than the customers' WTP values, then the customers will buy the products or services. Thus, there is a direct connection between payment intention and WTP values (Castellanos et al., 2011; Wicker et al., 2016). Based on the previous studies, this study hypothesizes:

**H5**. Users' payment intention to buy or subscribe to livestream sports broadcasts online will have a positive effect on WTP values.

### Summary of Study Goals

**Figure 1:**
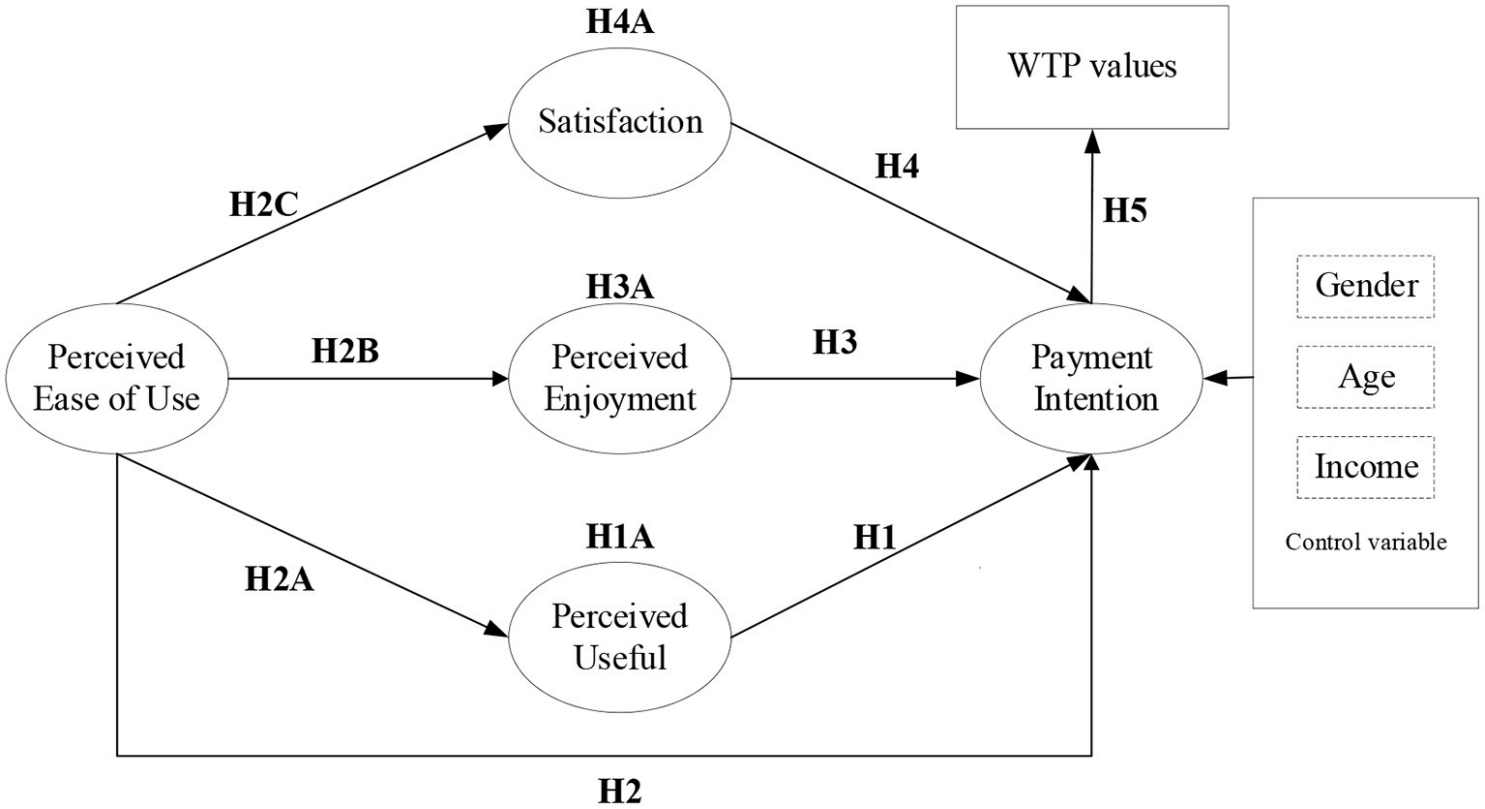
Conceptual model.

Although livestream network platforms have become an inevitable trend in sports broadcasting, the factors that affect users' willingness to pay for the new services are not yet clear. By integrating the TAM with the WTP, this study considered that perceived usefulness and perceived ease of use in the original TAM would directly relate to intention to pay. New variables (perceived enjoyment and satisfaction) were introduced to modify the TAM to test the determinants of pay intention for livestream sports platforms, while the relationship between perceived usefulness and perceived ease of use remained. Users with a high degree of perceived ease of use are expected to increase their satisfaction with the platforms, while perceived ease of use is expected to influence perceived usefulness. In addition, it is hypothesized that users who handle the livestream sports platforms easily, will find them to be more enjoyable. Perceived usefulness, perceived enjoyment, and satisfaction are expected to mediate between perceived ease of use and payment intention. Based on the analysis thereof, the full conceptual model is illustrated in Figure 1.

## Method

### Data Collection

To protect the privacy of participants, the data were collected by distributing online-questionnaires in China. All questionnaires were using an anonymous survey. The study was approved by the Institutional Review Board of the Renmin University School of Journalism and Communication.

The significant premise of variables in this study was that users have previously watched sports events through network broadcast platforms, and therefore, all the participants were first asked, whether they had ever used livestream broadcast platforms to watch sports events. If their answer was “no” they did not continue with the survey and were excused from the study. Respondents who answered, “yes,” were asked to participants in the survey. In total, 377 individuals responded to the online-questionnaires, and 330 (87.5%) of them successfully completed all of the scales.

According to the demographic data, 15.8% of the respondents were females, and 84.2% were male. In terms of respondents' ages, 26–34 years-old accounted for the highest proportion, reaching 48.8% of the total sample. Individuals aged 19–25 comprised of 33.3%, followed by 35–44-year-olds, at 14.8%. Respondents in this study under the age of 19 (0.6%) and over the age of 45 (2.4%) accounted for the lowest percentages. Regarding educational backgrounds, the largest group was comprised of undergraduates (67.7%), followed by high school graduates (17.9%), then postgraduates at 11.8%, followed by doctoral students representing only 2.7% of the sample. None of the respondents were educated at less than high school graduation.

### Measure

#### Perceived Usefulness

Using a seven-point Likert scale (1 = strongly disagree, 7 = strongly agree), three items from Davis et al. (1989) and Dickinger et al. (2008) were introduced to measure users' perceived usefulness (e.g., “I find watching livestream sports broadcasts through the Internet to be useful to me,” “Livestream sports broadcasts are valuable for my communication effectiveness”). The measure's Cronbach alpha coefficient was 0.84.

#### Perceived Ease of Use

Similar to the previous studies (Alalwan et al., 2018), perceived ease of use was measured by three items (e.g., “Watching livestream sports is clear and understandable,” “I find it is easy to watch livestream sports through Internet”) on a seven-point scale (1 = strongly disagree, 7 = strongly agree). The three-item perceived ease of use scale exhibited high levels of internal consistency (α = 0.84).

#### Perceived Enjoyment

Items from Abdullah et al. (2016) were adapted for hedonic system usage to form a 3-item measure to assess users' perceived enjoyment (e.g., “I have fun watching livestream sports broadcasts on the computer,” “I find watching livestream sports online enjoyable”). The items were answered on a 7-point Likert scale, with higher scores reflecting greater perceived enjoyment. The perceived enjoyment exhibited high internal consistency (α = 0.86).

#### Satisfaction

Satisfaction is regarded as consumer' overall evaluation when they find that products or services meet or exceed their positive expectations (Santini et al., 2018; Chung et al., 2020). Therefore, degrees of satisfaction may be influenced by all components of a service or product (Miranda et al., 2018). Based on a previous study (Zhang et al., 2006), three items were developed specifically for this study to assess users' satisfaction with livestream sports broadcasts (e.g., “I'm very satisfied with the commentary on the livestream sports broadcasts,” “I'm very satisfied with the clarity of the livestream sports broadcasts.”). All items were assessed on a seven-point Likert scale and combined in a scale (α = 0.83).

#### WTP and WTP Values

Drawing on previous studies (Arogundade et al., 2016), payment intention was assessed by three items (Park et al., 2007; Abdullah et al., 2016). The three-item payment intention scale (e.g., “Given that I had access to pay for live sports online, I predict that I would pay for it,” “I plan to pay for live sports online in the future”) had high internal consistency (α = 0.85). In addition, respondents about their maximum WTP: “Being more explicit, if you were willing to pay for livestream sports on the internet, what is the maximum monthly rate you would be willing to pay?”

## Results

Structural equation modeling (SEM) is the most efficient technique for estimating a series of separate multiple regressions (Hair et al., 2009). SEM primarily consists of two components; the measurement model and the structural model. It is common practice to adopt SEM for analyzing the cause-effect relationship between latent constructs in the study of TAM (Lemay et al., 2018). All calculations were completed by Mplus 8.3 software. To verify that scales represented the intended constructs, the measurement model was fitted to the data, using confirmatory factor analysis (see Table 2).

**Table 2:** Confirmatory factor analysis (CFA).

**Scales**	**Items**	**Factor loading**	***P*-value**	**Composite reliability**
Perceived	PU1	0.778	0.000	0.840
usefulness	PU2	0.809	0.000	
	PU3	0.806	0.000	
Perceived	PE2	0.827	0.000	0.845
ease of use	PE3	0.751	0.000	
	PE4	0.829	0.000	
Satisfaction	SAT1	0.779	0.000	0.833
	SAT2	0.807	0.000	
	SAT3	0.785	0.000	
Perceived	PEJ1	0.774	0.000	0.865
enjoyment	PEJ2	0.757	0.000	
	PEJ3	0.937	0.000	
Payment	PI1	0.806	0.000	0.852
intention	PI2	0.852	0.000	
	PI3	0.772	0.000	

As shown in Table 3, the AVE for all variables in this study are above the recommended value of 0.5 and reach above 0.6. Therefore, convergent validity is satisfactory. The bold number on the diagonal are the square of the AVE. And all the bold numbers (0.803, 0.779, 0.826, 0.797, 0.810) exceeded the latent construct's highest correlation with any other latent construct, thus demonstrating that the underlying variables exhibited the property of discriminant validity.

**Table 3:** Convergent validity and discriminant validity.

**DIM**.	**Convergence validity**	**Discriminate validity**				
	**AVE**	**PU**	**PE**	**PEJ**	**SAT**	**PI**
Perceived usefulness	0.645	**0.803**				
Perceived ease of use	0.607	0.271	**0.779**			
Perceived enjoyment	0.683	0.367	0.172	**0.826**		
Satisfaction	0.636	0.466	0.186	0.335	**0.797**	
Payment intention	0.656	0.258	0.037	0.319	0.268	**0.810**

*The bold number on the diagonal are the square of the AVE*.

*The bold number exceeded the latent construct's highest correlation with any other latent construct*.

*The AVE for all variables in this study are above the recommended value of 0.5*.

The model fit indices were good (see Table 4; χ = 282.875, df = 142, χ/df = 1.992, CFI = 0.943, TLI = 0.933, RMSEA = 0.055, SRMR = 0.083).

**Table 4:** Model validation.

**Goodness-of-fit**	**χ**	***df***	**χ^2^/*df***	**CFI**	**TLI**	**RMSEA**	**SRMR**
Recommended Value	-	-	1 < χ2/df <3	>0.9	>0.9	<0.08	<0.10
Overall Model	282.875^***^	142^***^	1.992	0.943	0.933	0.055	0.083
Improved Model	216.355^***^	113^***^	1.915	0.958	0.949	0.053	0.083
Whether meet standards	-	-	YES	YES	YES	YES	YES

*** *P < 0.001*.

### Direct Effect on Payment Intention and WTP Values

The SEM was conducted to test the proposed hypotheses. The findings indicated that three hypotheses regarding direct effect on payment intention and WTP values were supported, and two hypotheses were not support at a statistically significant level (see Table 5).

**Table 5:** Hypotheses test.

**DV → IV**	**Point estimate**	**Product of coefficient**		**BOOTSTRAP 1000 TIME 95% CI**				
				**Bias corrected**		**Percentile**		
		**S.E**.	**Z**.	**Lower**	**Upper**	**Lower**	**Upper**	***P*-value**
**STANDARDIZED DIRECT EFFECTS**								
H1: PU → PI	0.057	0.068	0.838	−0.084	0.181	−0.076	0.188	0.402
H2: PE → PI	−0.003	0.067	−0.043	−0.137	0.130	−0.137	0.130	0.966
H3: PEJ → PI	0.158	0.056	2.816	0.046	0.269	0.046	0.268	^**^
H4: SAT → PI	0.264	0.068	3.901	0.133	0.401	0.135	0.401	^***^
H5: PI–WTP VALUES	4.268	0.794	5.374	2.960	6.161	2.884	6.015	^***^
H2A:PE → PU	0.450	0.062	7.251	0.334	0.570	0.330	0.568	^***^
H2B:PE → PEJ	0.378	0.067	5.602	0.260	0.523	0.255	0.517	^***^
H2C:PE → SAT	0.462	0.069	6.653	0.348	0.611	0.332	0.601	^***^
**STANDARDIZED INDIRECT EFFECTS**								
H1A:PE → PU → PI	0.026	0.031	0.838	−0.033	0.083	−0.033	0.084	0.402
H3A:PE → PEJ → PI	0.060	0.023	2.601	0.021	0.109	0.018	0.106	^**^
H4A:PE → SAT → PI	0.122	0.035	3.485	0.065	0.207	0.060	0.200	^***^

** *P < 0.001*,

*** *P < 0.01*.

H3 and H4 suggested that perceived enjoyment (β = 0.158, *p* < 0.001), satisfactorily (β = 0.264, *p* < 0.01) had positive, significant, and direct effects on willingness to pay for livestream sports broadcasts on the internet. The fifth hypothesis, which examined the relationship between the willingness to pay and the WTP value: users' payment intention (β = 4.268, *p* < 0.001) to purchase or subscribe to livestream sports online is positively related to the WTP value.

Perceived usefulness (β = 0.057, *p* = 0.402) had no effect on users' payment intention to pay for or subscribe to livestream sports online. Similarly, there was no significant relationship between perceived ease of use (β = −0.003, *p* = 0.966) and the willingness to pay for livestream sports online. Thus, H1 and H2 were not supported.

### Direct Effect of Perceived Ease of Use

Perceived ease of use, a fundamental external motivation variable, exhibited a crucial role in the study of willingness to pay for livestream sports broadcasts online. The result confirmed that perceived ease of use (β = 0.450, *p* < 0.001) positively affected perceived usefulness, and supported H2A. Perceived ease of use (β = 0.378, *p* < 0.001) had a positive and significant influence on users' perceived enjoyment, supporting H2B. H2C examined the relationship between perceived ease of use and satisfaction, suggesting users' perceived ease of use (β = 0.462, *p* < 0.001) was positively associated with satisfaction.

### Moderating Role of Perceived Enjoyment and Satisfaction

**Figure 2:**
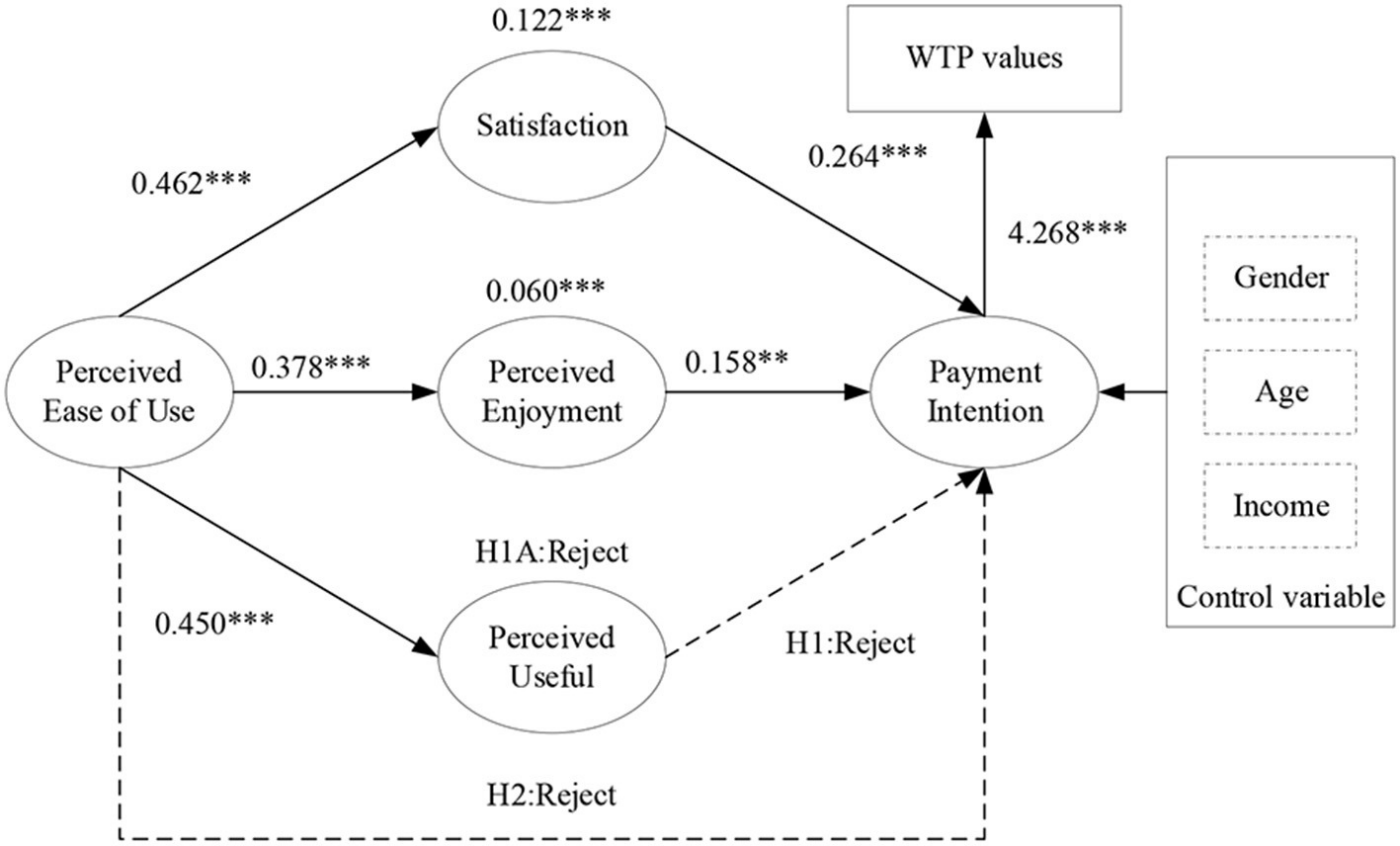
Result model. **p < 0.001, ***p < 0.01.

The finding of H3A indicated that perceived ease of use (β = 0.060, *p* < 0.01) had a significant, albeit indirect influence on the willingness to pay for livestream sports broadcasts online via the mediator of perceived enjoyment. H4A confirmed that satisfaction mediated the effect of perceived ease of use on users' intention to buy or subscribe to livestream sports online (β = 0.122, *p* < 0.001). The indirect effect of perceived ease of use on payment intention to buy or subscribe to livestream sports online by way of perceived usefulness was insignificant (β = 0.026, *p* = 0.402), rejecting H1A. The result model was shown on Figure 2.

Ruling out the variables that were insignificant to the willingness to pay for livestream sports online, the modified model vastly improved the evaluation processes of the former model (χ^2^ = 216.355, df = 113, χ^2^/df = 1.915, CFI = 0.958, TLI = 0.949, RMSEA = 0.053, SRMR = 0.083).

## Discussion

As live streaming of sporting events has become essential to fans and networks, understanding users' willingness to pay (WTP) has become vital. Combining perceived enjoyment, satisfaction with the Technology Acceptance Model (TAM), this study combined information system research with the consumer demand theory to examine factors influencing users' motivations to stream online sports. The rise of livestream sports broadcasts online is based on video streaming technologies, and required the establishment of a new model that could identify factors that influence users' payment intention based on external and internal motivations of technology acceptance. Perceived usefulness, perceived enjoyment, and satisfaction were positively related to the intention to pay for livestream sports broadcasts online. Congruent with previous studies (Van der Heijden, 2003; Abed, 2016), the results of this study supported the hypothesis that perceived enjoyment has a positive effect on users' payment intentions, and further confirmed that perceived enjoyment is the most significant factor in motivating payment intention. The impact of users' perceived enjoyment of livestream sports broadcasts for pay appear to be stronger when there are no free broadcast alternatives unavailable.

The direct connection between satisfaction and payment intention proved to be slightly stronger than the connection between perceived enjoyment and intention to pay for livestream sports broadcasts. The reason for the result might be that satisfaction is an emotional motivation based on repeated experiences. For hedonic systems, satisfied users are likely to be more significantly affected by positive emotional experiences that would lead to an increase in willingness to pay (Eisenbeiss et al., 2014; Tudoran and Olsen, 2017). Furthermore, satisfaction involves users' evaluations of clarity, speed, and commentary (Zhang et al., 2006) of livestream sports broadcasts in this study. The results suggested that users generated the willingness to pay primarily depending upon “their overall appraisal of how well they felt throughout the process of” using the service (Lee and Lehto, 2013).

Contrary to the hypothesis, the association between perceived ease of use and payment intention was not significant, which was consistent with the findings of previous research (Lee and Lehto, 2013). A reasonable explanation for the insignificant finding might be that it is very easy for users to watch sports events through live network platforms and realize real-time interaction with other spectators by “bullet comment.” Another plausible explanation may be that ease of use proves to be irrelevant to users since instrumentality is prioritized over ease of use (Nagy, 2018). In sum, the users may not pay merely for the ease of use for livestream sports broadcasts. However, perceived enjoyment and satisfaction exerted complete mediating roles in the relationship between perceived ease of use and intention. The results of the mediation model were consistent with previous studies (Park et al., 2007; Pai and Huang, 2011), which suggested that the greater users' perceived ease of use to be, the more their satisfaction and perceived enjoyment improved, increasing their willingness to pay for livestream sports broadcasts.

Interestingly, perceived usefulness had no statistically significant effect on willingness to pay for livestream sports broadcasts. Previous studies on other hedonic products, such as an online game, confirmed the same finding (Wu and Zhou, 2017). A more plausible explanation would be that intrinsic motivation has a more dominant effect than extrinsic motivation, and especially in the hedonic system, extrinsic motivations may not exist (Wang and Scheepers, 2012). Users watch online sports events via the Internet during leisure time for fun, rather than for utilitarian-oriented usefulness.

## Conclusions

This study not only enhanced the technology acceptance model by introducing two variables, perceived enjoyment (Vladova et al., 2021) and satisfaction (You et al., 2020; Liu et al., 2021) in the original TAM, but also integrate TAM and WTP to explicit the factors affecting users' payment intention to online sports streaming. It was confirmed that satisfaction has a positive effect on payment intention to buy or subscribe to livestream sports broadcasts. The findings of questionnaire indicated that all the respondents are willing to pay 16.1 yuan ($2.43) a month on average for online sports streaming. From the perspective of practice, this study provides significant evidence for the live football online platforms to take measures to improve user payment in the future, and the user experiences might be improved.

The significance of perceived enjoyment is emphasized in this study. Therefore, the business strategies of improving users' perceived enjoyment are an effective method to promote users' willingness to pay. In consideration of prior researches on hedonic products, it might be a good idea to promote live football online platforms with movies (Wang and Scheepers, 2012) or short video, as both of them have the same function with football live online. In addition, platforms should establish an interactive mechanism such as increasing the interest of “bullet comment” that users exchange ideas about football matches to strengthening interest and relax. There is no doubt that “bullet screen” play a vital role in meeting the entertainment needs of users (Tong and Zhao, 2019). However, it is worth noting that users can send “bullet comment” anonymously, which aggravates the cyber risk. In practice, cyber-crime is hidden in the “bullet screen”-criminal organizations induce users to access the illegal sports gambling websites, stealing private information of users by injecting malicious script code in web pages and causing the users' financial losses by network attacks.

Equally important, the study results confirm that satisfaction have a positive effect on payment intention. Therefore, how to improve user satisfaction to promote existing users' payment intention and attract more new users is one of the crucial issues for live sports online platforms. For instance, the platforms should invest more capital in technological innovation to improve digital images definition and operating speed, while the football commentators should enhance the specialized quality, change the language style to fit in with the needs of the internet. Due to the outright cancellation or postponement of major events impacted by COVID-19 outbreak, it increases the operating risks of online sports streaming. Therefore, streaming media can increase highlights, short-form content and athlete-generated content instead of live video content (PwC, 2020) to upgrade users' satisfaction during the lockdown.

Our findings indicate that users' intentions to pay for sports live online are influenced by perceived enjoyment and satisfaction. This provides a good starting point for discussion and further research. But the findings of study have to be seen in light of some limitations.

First, the sample was cross-sectional, which limited the ability to make causal inference. It is a dynamic and continuous process form users' payment intention to their actual purchase behavior, and then to the continuous intention of purchasing a product (Liu et al., 2021). Longitudinal study is warranted for future studies.

Secondly, the analysis of WTP that all participants were users in China, the empirical findings of this study may not generalize to users in other nations. Different countries have different levels of development of the live football online platforms, while users in other countries have different payment behaviors. Future studies should expand the effective range of samples and the quantity with a wider geographical area (Tsai et al., 2020; Carranza et al., 2021).

Thirdly, cyber risk is the objective risk caused by streaming media technology. But whether users can perceive the cyber risk subjectively is also an important topic. Therefore, future research could categorize cyber risk of online sports streaming media into data risk, anonymous risk, cyber-crime risk, hacker risk (Sheehan et al., 2021) to explore users' perception of cyber risk, and further confirm the relationship between perceived cyber risk and willingness to pay of users.

## Data Availability Statement

The raw data supporting the conclusions of this article will be made available by the authors, without undue reservation.

## Ethics Statement

The studies involving human participants were reviewed and approved by Renmin University of China, School of Journalism, and Communication, Institutional Review Board. The participants provided their informed consent to participate in this study.

## Author Contributions

All authors listed have made a substantial, direct and intellectual contribution to the work, and approved it for publication.

## Conflict of Interest

The authors declare that the research was conducted in the absence of any commercial or financial relationships that could be construed as a potential conflict of interest.
